# Premature atrial and ventricular complexes in outpatients referred from a primary care facility

**DOI:** 10.1371/journal.pone.0204246

**Published:** 2018-09-20

**Authors:** Wilma Noia Ribeiro, Alice Tatsuko Yamada, Cesar José Grupi, Gisela Tunes da Silva, Alfredo Jose Mansur

**Affiliations:** 1 Heart Institute, University of São Paulo Medical School, São Paulo, Brazil; 2 Institute of Mathematics and Statistics, University of São Paulo, São Paulo, Brazil; University of Tampere, FINLAND

## Abstract

**Background:**

Premature complexes are common electrocardiographic findings in daily clinical practice that require further evaluation. Investigation may sometimes be complex and expensive. The aim of our study was to analyze variables associated with premature beats identified in outpatients referred from a primary care facility.

**Materials and methods:**

We performed a cross-sectional study of 407 outpatients (aged 55.8±11years; 56% women) who were followed by general practitioners and were referred for resting 12-lead electrocardiograms for a routine clinical follow-up. After signing informed consent, patients answered a questionnaire and underwent physical examinations, laboratory diagnostics, transthoracic echocardiograms and 24-hour Holter monitoring to evaluate for the presence of premature complexes. After the univariate analyses, logistic regression analyses were performed with adjustment for age, sex, and cardiovascular diseases.

**Results:**

Premature complexes distribution revealed that they were frequent but with low density. Premature atrial complexes (≥ 4/hours) were associated with age (Odds Ratio (OD) = 1.030, Confidence Interval (CI) 95% = 1.002 ─ 1.059, p = 0.029), brain natriuretic peptide (BNP) levels > 20mg/dL (OR = 4.489, 95%CI = 1.918 ─ 10.507, p = 0.0005), intraventricular blocks (OR = 4.184, 95%CI = 1.816 ─ 9.406, p = 0.0005) and left atrial diameter (OR = 1.065, 95%CI = 1.001 ─ 1.134, p = 0.046). Premature ventricular complexes (≥ 5/hour) were related to age (OR = 1.032, 95%CI = 1.010 ─ 1.054, p = 0.004), the use of calcium channel blockers (OR = 2.248, 95%CI = 1.019 ─ 4.954, p = 0.045), HDL-cholesterol levels (OR = 0.971, 95%CI = 0.951 ─ 0.992, p = 0.007), BNP levels > 20mg/dL (OR = 2.079, 95%CI = 0.991 ─ 0.998, p = 0.033), heart rate (OR = 1.019, 95%CI = 1.001 ─ 1.038, p = 0.041), left ventricular hypertrophy (OR = 2.292, 95%CI = 1.402 ─ 3.746, p = 0.001) and left ventricular ejection fraction (OR = 0.938, 95%CI = 0.900 ─ 0.978, p = 0.002).

**Conclusions:**

Premature complexes had low density and were associated with BNP levels > 20mg/dL, lower levels of HDL-cholesterol, left atrial enlargement and ventricular hypertrophy. The identification of premature complexes on 24-hour Holter monitor recordings of outpatients in a primary public healthcare setting was associated with uncontrolled cardiovascular risk factors that may be addressed with medical advice and therapy in a primary care setting.

## Introduction

Extrasystoles are everyday findings on clinical examinations or electrocardiographic records of outpatients referred from primary care facilities and may be either asymptomatic or symptomatic. While they may be benign in nature, evaluation of their clinical implications and potential association with other clinical variables is necessary.

Variables associated with extrasystoles seem to differ according to the characteristics of the studied population. Premature atrial complexes identified in the 24-hour Holter monitor recordings of 1742 healthy Swiss adults were associated with age (odds ratio = 1.80; confidence interval 95% = 1.60–2.02; p<0.0001), previous cardiovascular disease (odds ratio = 2.40; confidence interval 95% = 1.64–3.51; p<0.0001), physical activity (odds ratio = 0.69; confidence interval 95% = 0.54–0.87; p = 0.002), HDL-cholesterol levels (odds ratio = 0.8; confidence interval 95% = 0.71–0.90; p = 0.0002) and N-terminal pro B-type natriuretic peptide levels (odds ratio = 1.27; confidence interval 95% = 1.13–1.41; p<0.0001)[1].

Premature ventricular complexes detected on the 24-hour Holter monitor recordings of 2048 young adults (mean age of 37 years) from Liechtenstein were associated with smoking (odds ratio = 2.18; confidence interval 95% = 1.21–4.01; p = 0.01), physical activity (odds ratio 0.51; confidence interval 95% = 0.34–0.76; p<0.001), N-terminal pro B-type natriuretic peptide levels (odds ratio = 1.52; confidence interval 95% = 1.30–1.76; p<0.01) and left ventricular hypertrophy (odds ratio = 1.38; confidence interval 95% = 1.15–1.66; p<0.01) [2].

Premature complexes are also frequently identified in our daily practice, and this prompted us to study the variables associated with these findings in outpatients who were followed by general practitioners in a primary public healthcare setting in São Paulo, Brazil.

Therefore, the aim of the present study was to assess the clinical and laboratory variables, including serum cholesterol and brain natriuretic peptide (BNP), associated with premature complexes identified on 24-hour Holter monitor recordings of outpatients referred from primary care facilities in São Paulo, Brazil.

## Materials and methods

### a) Patients

We performed a cross-sectional study of outpatients who were being followed by general practitioners at primary healthcare facilities and who were referred for a resting 12-lead electrocardiogram, usually as part of a clinical routine. For every two patients who had premature complexes identified on the 12-lead electrocardiogram, the next patient without an arrhythmia was invited to participate in the investigation. These outpatients answered a questionnaire and underwent a physical examination, fasting laboratory testing, a transthoracic echocardiogram and 24-hour Holter monitoring within five days after enrollment.

Examinations were performed at the Doctor Fernando Mauro Pires da Rocha Municipal Hospital, which is a secondary care public hospital in the south region of São Paulo City in the district of Campo Limpo (“Clean Field”). This district has 222 301 inhabitants (relative to 11.7 million of the City) [3] and a human development index of 0.806 [4].

The São Paulo public health system is divided into regions and comprises several hospitals surrounded by basic health care units. The Doctor Fernando Mauro Pires da Rocha Municipal Hospital is the referral hospital for 25 basic health care units in the district of Campo Limpo [3].

The inclusion criteria were outpatients older than 18 years who underwent 24-hour Holter monitoring between January 2009 and December 2012.

The exclusion criteria were a permanent pacemaker, atrial fibrillation or flutter, second- or third-degree atrioventricular block, a history of acute coronary syndrome within 3 months and the use of antiarrhythmic drugs (apart from the use of beta-blockers for the treatment of arterial hypertension). Participants with Holter recordings shorter than 18 hours duration or of insufficient quality were also excluded.

The protocol was approved by the institutional review boards of Hospital das Clínicas of São Paulo University Medical School (CAAE 27163514.6.0000.0068) and the Hospital Doctor Fernando Mauro Pires da Rocha / São Paulo City Health Secretary (number 31/06). Participants signed an informed consent before inclusion in the investigation. The data from our study are available from the Harvard database (accession number https://doi.org/10.7910/DVN/C2LS02).

### b) Clinical and laboratory data

Patients’ ages, symptoms, history of diseases, physical activities, smoking status, alcohol and caffeine consumption and current medications were obtained from a questionnaire. Body mass index was calculated by dividing the weight in kilograms by the square of the height in meters, and systolic and diastolic blood pressures were measured for each patient.

The presence of coronary artery disease was defined as a history of myocardial infarction, coronary artery bypass graft surgery, percutaneous coronary intervention, or the presence of an electrically inactive area on the 12-lead electrocardiogram.

Participants who were current smokers or who had stopped smoking less than one year before the study were classified as smokers, while the others were classified as nonsmokers. To standardize alcohol intake, the number of drinks per week was measured, with a standard dose representing 12 g of alcohol, as defined by the World Health Organization. We calculated the consumption of each beverage multiplied by its ethanol content (4.8 g for beer, 9.6 g for wine, and 32 g for liquor) and the total of all the beverages.

Exercise level was based on the "International Physical Activity Questionnaire" [5], and participants were classified as sedentary if they did not exercise or only engaged in low levels of physical activity. Subjects who engaged in moderate or high levels of exercise were classified as active people.

Blood samples were obtained after 12 hours of fasting to measure glucose, glycosylated hemoglobin, magnesium, potassium, creatinine, uric acid, brain natriuretic peptide (BNP), thyroid-stimulating hormone, total cholesterol, HDL-cholesterol, and triglycerides levels and to perform the serology for Chagas’ disease. LDL-cholesterol was calculated using the Friedewald equation [6] (all in mg/dL) for subjects who had triglyceride levels ≤ 400mg/dL, or was obtained directly. Creatinine clearance was calculated using the Cockcroft-Gault equation [7].

The variables from the 12-lead electrocardiogram that were studied included heart rate, and the presence of intraventricular blocks, left atrial overload, left ventricular overload and electrically inactive areas [8].

Participants also underwent a two-dimensional transthoracic echocardiographic examination. A cardiologist who was blinded to the patients’ clinical information and laboratory results interpreted these exams according to the recommendations of the American Society of Echocardiography Committee [9].

Echocardiographic variables included left atrial diameter, aortic diameter, septal thickness, posterior wall thickness, left ventricular diastolic and systolic diameters, the presence of diastolic dysfunction or valvular disease, and left ventricular ejection fraction, which was derived from a 2-dimensional measurement or M-mode. Left ventricular mass was calculated using the Devereux equation [10] and indexed by dividing by body surface area.

### c) Holter recording

Ambulatory 24-hour electrocardiographic monitoring was performed. Three-lead monitoring was used, and the recordings were manually reviewed by a cardiologist to ensure accuracy. Patients were advised to perform their routine everyday activities normally and register their daily activities in a diary.

The mean number of premature complexes per hour was used as the primary outcome in our study, and the cutoff points for premature atrial and ventricular complexes were four and five per hour, respectively. The choice of these values was based on recent studies that revealed that lower frequencies of premature complexes/hour (approximately 100/day) were associated with a worse prognosis [11,12].

Since these arrhythmias can share similar associations, the control group included patients with < 4 premature atrial complexes/hour and < 5 premature ventricular complexes/hour.

Symptoms, mean heart rate and QT-interval duration on the 24-Holter monitor recordings were also evaluated on our study.

### d) Statistical analysis

The results are expressed as percentages for categorical variables and as the mean value ± standard deviation for continuous variables. Baseline characteristics were compared using Student’s *t*-test for continuous variables and the chi-squared test for categorical variables.

Multivariate analysis was performed using the logistic regression method [13] to identify independent variables associated with the presence of these arrhythmias. The analysis was divided into two parts: a logistic regression model in which we compared individuals in the control group with patients with frequent premature atrial complexes and a second model that compared individuals in the control group with patients with frequent premature ventricular complexes.

The methodology used was the same for both models: a full model was considered, and a selection procedure was applied in order to derive a final model that included only the significant factors. The full model included all the variables that had a significance < 0.10 in the preliminary univariate analysis in addition to the fixed variables including age, sex, history of arterial hypertension and coronary artery disease, use of statins and BNP, HDL-cholesterol, and LDL-cholesterol levels.

The selection of independent variables was performed in one step; a backward procedure was applied to the full model, and the variables were eliminated in order to generate a final model including only those variables that were significant at a level of 0.05.

Receiver operating characteristic (ROC) curves were constructed for premature atrial and ventricular complexes, and the best cutoff value was calculated for each continuous variable that showed an independent association with ectopic beats in the logistic regression.

The statistical analysis was performed using the software R, version 3.3.1. A two-tailed p-value < 0.05 was prespecified to indicate statistical significance.

## Results

Four hundred and seven outpatients were evaluated in our study. The mean age was 55.8±11years, and 228 subjects (56%) were women. A history of arterial hypertension was observed in 288 (71%) participants, diabetes mellitus in 89 (22%) participants, dyslipidemia in 70 (17%) participants, and coronary artery disease in nine (2%) patients. The mean left ventricle ejection fraction was 68±5%, and the mean BNP level was 19.5±23mg/dL.

Eight patients with atrial fibrillation, 12 participants who were taking antiarrhythmic drugs and nine patients with recent acute coronary syndrome were excluded. Six patients had Holter monitor recordings < 18 hours’ duration, and 12 had recordings that had insufficient quality. Therefore, 47 outpatients were excluded from our analysis.

**Fig 1:**
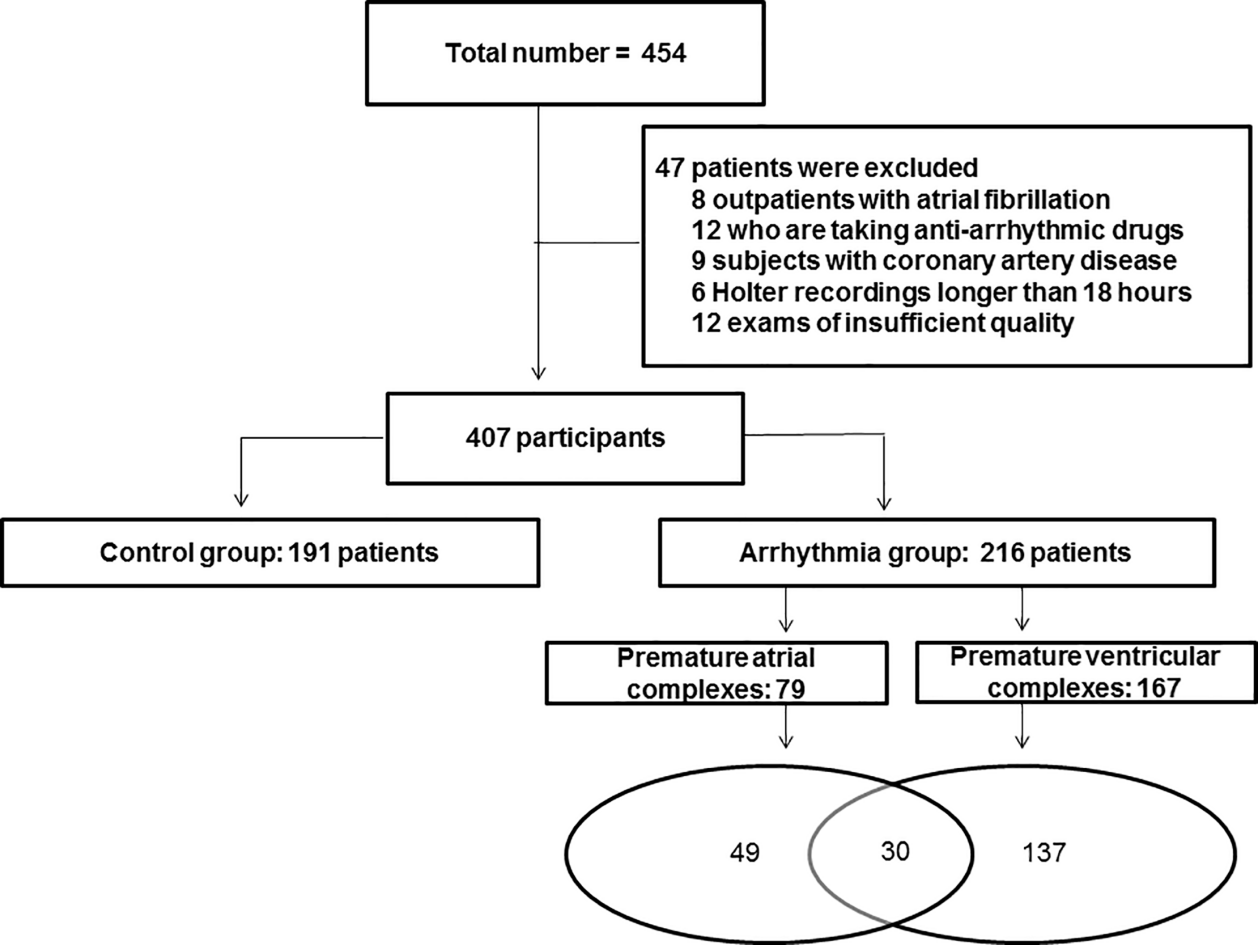
Flowchart showing the classification of patients.

The average duration of the recordings was 22.6 ± 1.5 hours, and 41 patients (10% of our sample) did not have any premature complexes on the 24-hour Holter monitor recordings. A flowchart presenting the classification of patients according to the frequency of premature contractions is shown in Fig 1.

### a) Premature atrial complexes

Seventy-nine outpatients with ≥ 4 premature atrial complexes/hour (29%) and 191 participants with < 4 premature atrial complexes/hour (71%) were evaluated. Outpatients with ≥ 4 premature atrial complexes were significantly older and used more angiotensin-converting enzyme inhibitors or angiotensin receptor blockers for the treatment of arterial hypertension than those who had fewer than 4 premature atrial complexes (Table 1). In the analysis of the laboratory variables, the group with frequent premature complexes had higher BNP levels (Table 2), and intraventricular blocks were also observed more frequently in this group (Table 3). These patients with frequent premature complexes also had larger left atrial diameter, septal and posterior wall thickness, and ventricular mass index on echocardiography (Table 3).

**Table 1:** Clinical characteristics according to the presence of premature atrial and ventricular complexes.

	Control group	Premature atrial complexes (≥ 4/h)		Premature ventricular complexes (≥ 5/h)	
	N = 191	N = 79	p-value	N = 167	p-value
**Age (years)**	53.7 (±11)	60.2 (±12)	0.0001	57.7 (±10)	0.0007
**Female sex, n (%)**	102 (53%)	45 (57%)	0.69	99 (59%)	0.31
**Palpitations, n (%)**	73 (38%)	28 (36%)	0.86	66 (40%)	0.85
**Hypertension, n (%)**	128 (67%)	63 (80%)	0.09	121 (72%)	0.35
**Diabetes mellitus, n (%)**	37 (19%)	19 (24%)	0.49	40 (24%)	0.36
**Dyslipidemia, n (%)**	28 (15%)	16 (20%)	0.34	34 (20%)	0.19
**Coronary artery disease, n (%)**	6 (3%)	2 (3%)	1.00	2 (1%)	0.38
**Current smoking status, n (%)**	47 (25%)	11 (14%)	0.07	28 (17%)	0.09
**Physical activity, n (%)**	29 (15%)	12 (15%)	1.00	28 (17%)	0.79
**Alcohol consumption, drinks/week**	1.27 (±4.1)	2.32 (±8.2)	0.28	0.70 (±2.6)	0.12
**Caffeine consumption, mL/day**	249.9 (±303)	306.2 (±366)	0.23	290.2 (±337)	0.24
**Diuretics, n (%)**	68 (36%)	33 (42%)	0.42	77 (46%)	0.05
ACEI / ARB, n (%)*	78 (41%)	50 (63%)	0.001	77 (46%)	0.34
**Beta-blockers, n (%)**	32 (17%)	10 (13%)	0.51	25 (15%)	0.77
**Calcium channel blockers, n (%)**	13 (7%)	10 (13%)	0.18	23 (14%)	0.04
**Statins, n (%)**	5 (3%)	7 (9%)	0.05	9 (5%)	0.28
**Body mass index, kg/m**^**2**^	27.0 (±4.7)	27.5 (±5.1)	0.51	28.1 (±5.4)	0.04
**Systolic blood pressure, mmHg**	128.8(±15.6)	132.2(±21.7)	0.24	130.3 (±16.5)	0.40
**Diastolic blood pressure, mmHg**	83.1 (±10.1)	82.4 (±12.8)	0.68	82.9 (±9.9)	0.89

* ACEI/ARB = angiotensin-converting enzyme inhibitors/angiotensin receptor blockers

**Table 2:** Laboratory variables in outpatients with frequent premature complexes.

	Control group	Premature atrial complexes (≥ 4/h)		Premature ventricular complexes (≥ 5/h)	
	N = 191	N = 79	p-value	N = 167	p-value
**Fasting glucose, mg/dL**	111 (±44)	109 (±42)	0.84	111 (±39)	0.99
**Glycosylated hemoglobin, %**	5.98 (±1.1)	5.99 (±0.8)	0.87	6.08 (±1.2)	0.39
**Total cholesterol, mg/dL**	200.1 (±40)	197.4 (±38)	0.61	197.8 (±38)	0.59
**HDL-cholesterol, mg/dL**	48.1 (±15)	47.2 (±11)	0.60	45.7 (±11)	0.08
**LDL-cholesterol, mg/dL**	120.3 (±36)	123.5 (±33)	0.49	126.0 (±33)	0.12
**Triglycerides, mg/dL**	152.9 (±96)	137.7 (±82)	0.19	134.6 (±64)	0.04
**Serum creatinine, mg/dL**	0.93 (±0.24)	0.93 (±0.26)	0.87	0.93 (±0.22)	0.94
**Creatinine clearance, mL/min**	88.1 (±27)	81.2 (±28)	0.08	84.9 (±28)	0.32
**Brain natriuretic peptide, mg/dL**	16.6 (±16)	37.6 (±33)	0.0003	34.1 (±53)	0.004
**Uric acid, mg/dL**	5.3 (±1.4)	5.4 (±1.8)	0.81	5.4 (±1.6)	0.61
**Positive serology for Chagas’ disease**	7 (4%)	3 (4%)	1.00	8 (5%)	0.80
**Levels of magnesium**					
Normal	179 (96%)	75 (99%)	0.55	157 (97%)	0.91
High	1 (1%)	0 (0%)	-	1 (1%)	-
Low	6 (3%)	1 (1%)	-	4 (2%)	-
**Levels of potassium**					
Normal	184 (96%)	76 (98%)	0.97	161 (98%)	0.68
High	3 (2%)	1 (1%)	-	1 (1%)	-
Low	3 (2%)	1 (1%)	-	3 (2%)	-
**Levels of TSH** *					
Normal	173 (92%)	70 (91%)	0.97	152 (92%)	0.94
High	13 (7%)	5 (6%)	-	10 (6%)	-
Low	1 (1%)	2 (3%)	-	3 (2%)	-

* TSH = thyroid stimulating hormone

**Table 3:** Electrocardiographic, Holter, and echocardiographic variables in patients with frequent premature complexes.

*Electrocardiographic variables*	Control group	Premature atrial complexes (≥ 4/h)		Premature ventricular complexes (≥ 5/h)	
	N = 191	N = 79	p-value	N = 167	p-value
**Heart rate, bpm**	73 (±13)	73 (±12)	0.95	75 (±12)	0.03
**Left atrial overload, n (%)**	49 (26%)	29 (37%)	0.09	69 (41%)	0.002
**Left ventricular overload, n (%)**	10 (5%)	7 (9%)	0.40	18 (11%)	0.08
**Intraventricular blocks, n (%)**	14 (7%)	22 (28%)	0.0001	28 (17%)	0.01
**Electrically inactive areas, n (%)**	5 (3%)	2 (3%)	1.00	2 (1%)	0.56
*Holter variables*					
**Mean heart rate, bpm**	78.8 (±12)	77.8 (±12)	0.51	78.7 (±11)	0.91
**QT interval, msec**	429.9 (±59)	437.5 (±57)	0.36	444.1 (±47)	0.02
**Record of symptoms, n (%)**	33 (24%)	19 (35%)	0.19	32 (26%)	0.85
*Echocardiographic variables*					
**Left atrial diameter, mm**	35.0 (±4.4)	36.8 (± 5.1)	0.01	36.3 (±5.4)	0.02
**Septal thickness, mm**	9.5 (±1.6)	10.1 (±1.5)	0.004	9.9 (±1.5)	0.008
**Posterior wall thickness, mm**	9.4 (±1.5)	9.9 (±1.5)	0.004	9.8 (±1.5)	0.005
**Diastolic diameter, mm**	48.9 (±4.9)	50.1 (±5.1)	0.09	51.0 (±5.3)	0.0002
**Systolic diameter, mm**	29.9 (±3.9)	30.8 (±4.6)	0.17	31.9 (±4.9)	0.0001
**Diastolic dysfunction, n (%)**	130 (70%)	59 (78%)	0.29	124 (76%)	0.32
**Valvular disease, n (%)**	2 (1%)	3 (4%)	0.30	6 (4%)	0.21
**Left ventricular ejection fraction, %**	68.7 (±5.4)	68.3 (±5.7)	0.59	66.8 (±6.2)	0.003
**Left ventricular mass index, g/m2**	97.1 (±27.4)	112.0 (±32.9)	0.001	110.0 (±29.9)	0.0001

With regard to the multivariate analysis, the following variables were categorized: creatinine clearance (cutoff value: 60mL/min) and left ventricular mass index (women at 95g/m^2^ and men at 115g/m^2^). Since the mean BNP value in our sample was 19.5mg/dL, for this variable, a cutoff value of 20mg/dL was used. The variables that were independently related to the presence of frequent premature complexes were age, BNP level, intraventricular blocks and left atrial diameter (Table 4).

**Table 4:** Multivariate model to assess the variables correlated with premature atrial complexes.

	Odds ratio	95% Confidence interval	P-value
**Age**	1.030	1.002–1.059	0.029
**Sex (female : male)**	1.175	0.598–2.307	0.640
**Hypertension**	0.623	0.241–1.605	0.327
**Coronary artery disease**	0.568	0.096–3.362	0.533
**ACEI / ARB** *	1.602	0.867–2.959	0.132
**Statins**	3.593	0.943–13.648	0.061
**HDL-cholesterol**	0.998	0.974–1.024	0.905
**LDL-cholesterol**	1.001	0.991–1.010	0.929
**Brain natriuretic peptide > 20mg/dL**	4.489	1.918–10.507	0.0005
**Creatinine Clearance > 60mL/min**	0.612	0.248–1.509	0.286
**Intraventricular blocks**	4.184	1.861–9.406	0.0005
**Left atrial diameter**	1.065	1.001–1.134	0.046
**Ventricular hypertrophy**†	1.394	0.727–2.673	0.318

* ACEI/ARB = angiotensin-converting enzyme inhibitors/angiotensin receptor blockers

† Ventricular mass index in women > 95gr/m^2^ and men > 115gr/m^2^

According to the ROC curve analysis, the best cutoff value for age was 67 years (sensitivity, 32.9%; specificity, 91.6%), and that for the left atrial diameter was 45 mm (sensitivity, 1.3%; specificity, 99.5%) for triggering premature atrial complexes.

### b) Premature ventricular complexes

We evaluated 167 outpatients with ≥ 5 premature ventricular complexes/hour (47%) and 191 outpatients with < 5 premature ventricular complexes/hour (53%); the results are shown in Tables 1, 2 and 3.

Participants with ≥ 5 premature ventricular complexes/hour were significantly older, had a higher body mass index and used more calcium channel blockers than those with less than 5 premature ventricular complexes/hour. This group of participants with more premature ventricular complexes had also lower triglyceride and higher BNP levels. With regard to the variables from the Holter monitor recordings and the 12-lead electrocardiograms, left atrial overload, intraventricular blocks, higher heart rate, and longer QT-interval were observed more frequently in patients with more premature ventricular complexes (≥ 5/hour). Larger left atrial diameter, septal and posterior wall thickness, left ventricular diastolic and systolic diameter, ventricular mass index, and lower left ventricular ejection fraction were found on the echocardiograms in the group with frequent ectopic beats.

In the multivariate analysis, the following variables were categorized: body mass index (30kg/m^2^), QT-interval (440msec), left ventricular mass index (women at 95g/m^2^ and men at 115g/m^2^) and BNP level (20mg/dL).

Premature ventricular complexes (≥ 5/hour) were independently associated with age, intake of calcium channels blockers, HDL-cholesterol, BNP level, heart rate, left ventricular hypertrophy and left ventricular ejection fraction (Table 5).

**Table 5:** Multivariate model to assess the variables correlated with premature ventricular complexes.

	Odds ratio	95% Confidence interval	P—value
**Age**	1.032	1.010–1.054	0.004
**Sex (female : male)**	0.859	0.509–1.453	0.572
**Hypertension**	0.747	0.421–1.326	0.319
**Coronary artery disease**	0.304	0.056–1.648	0.168
**Body mass index > 30kg/m**^**2**^	0.911	0.509–1.628	0.752
**Diuretics**	1.421	0.783–2.579	0.248
**Calcium channel blockers**	2.248	1.019–4.954	0.045
**Statins**	2.264	0.622–8.240	0.215
**HDL-cholesterol**	0.971	0.951–0.992	0.007
**LDL-cholesterol**	1.005	0.998–1.012	0.174
**Triglycerides**	0.995	0.991–1.010	0.002
**Brain natriuretic peptide > 20mg/dL**	2.079	1.062–4.068	0.033
**Heart rate**	1.019	1.001–1.038	0.041
**QT interval > 440 msec**	1.114	0.650–1.909	0.694
**Left atrial diameter**	1.038	0.986–1.094	0.153
Ventricular hypertrophy*	2.292	1.402–3.746	0.001
**Left ventricular ejection fraction**	0.938	0.900–0.978	0.002

* Ventricular mass index in women > 95gr/m^2^ and men > 115gr/m^2^

The ROC curve for age showed that outpatients > 65 years of age (sensitivity, 24.6%; specificity, 88%) had a higher prevalence of ectopic beats. The prevalence of premature complexes was also higher in outpatients who had HDL-cholesterol levels < 35mg/dL (sensitivity, 18.1%; specificity, 88.4%), heart rate > 79bpm (sensitivity, 30.5%; specificity, 78.5%) and left ventricular ejection fraction < 67% (sensitivity, 33.9%; specificity, 78.5%).

## Discussion

In this outpatient-based study, we found that 79 (19%) participants had ≥ 4 premature atrial complexes/hour, and 167 (41%) subjects had ≥ 5 premature ventricular complexes/hour on the 24-Holter monitor recordings.

Premature atrial and ventricular complexes were related to age in our study. The probability of occurrence of these arrhythmias for each year increment was approximately 3.0%, and the age from which these probabilities seemed to be higher was 67 years for premature atrial complexes and 65 years for premature ventricular complexes. Imbalances in the autonomic system and degeneration of the electric conduction system are important causes of the higher prevalence of premature complexes in the elderly.

We did not find an association between sex and ectopic beats in these outpatients, as in previous studies that analyzed these arrhythmias in the general population [1,2,14]. The mean age of the patients in our study sample was 55 years; age that is usually associated with decreasing testosterone and estrogen levels. These hormonal changes might have influenced the results we found.

An independent association between heart rate and frequent premature ventricular complexes was observed in our study, and this relationship was stronger in outpatients with heart rates > 79 bpm than in those with lower heart rates. This finding has been reported in previous studies [15,16], suggesting that activation of the sympathetic nervous system plays an important role in increases in heart rate and triggering of ectopic beats.

With regard to the use of antihypertensive medications, the use of calcium channel blockers was more prevalent in participants with frequent premature ventricular complexes, and the cause of this finding is not clear. The majority of these patients were using nifedipine at the time of enrollment in the study, and this medication causes peripheral vasodilation and reflex tachycardia due to increased autonomic activity. This effect could explain its association with premature ventricular complexes in our study and strengthens our finding that higher heart rates are related to this arrhythmia.

Premature ventricular complexes were more frequent in outpatients with left ventricular hypertrophy in our survey, as demonstrated in a previous study [17]. Ventricular hypertrophy is associated with an increase in the activity of the sympathetic nervous system and a progressive decrease in perfusion of the myocardium leading to ischemia and the formation of fibrotic areas, which are related to ventricular ectopic beats.

An association between premature atrial complexes and left atrial enlargement was observed, which was stronger when the left atrial size was > 45mm. We used a similar cutoff value to previous studies that found an association between this arrhythmia and atrial fibrillation (> 100/day) [11,18]. Once the association of left atrial size and atrial fibrillation is well known [19], the presence of both left atrial diameter > 45mm and frequent ectopic beats could help to identify outpatients with a higher risk of developing atrial fibrillation and thromboembolic events.

Our study demonstrated that hypertrophy and left atrial enlargement were associated with premature atrial and ventricular complexes, and as both conditions stimulate the release of BNP [20–22], we also found a relationship between this biomarker and these arrhythmias. This finding probably reflects an increased left ventricular filling pressure and atrial distension promoted by hypertrophy. Thus, the presence of BNP levels > 20mg/dL and premature complexes might identify a group of outpatients who would benefit from intensive treatment for arterial hypertension.

We did not find an increased frequency of arterial hypertension among participants with frequent premature complexes. As structural cardiac changes were found in these outpatients, such as left atrial enlargement and left ventricular hypertrophy, other causes of pressure overload might be operating in this setting.

We observed an inverse relationship between HDL-cholesterol and frequent premature ventricular complexes. There was a 2.9% reduction in the presence of this arrhythmia for each one-point increase in HDL-cholesterol, and this relationship was stronger when the level of this lipoprotein was < 35mg/dL. Only one survey has demonstrated an association between HDL-cholesterol and premature atrial complexes in the general population [1], and with regard to atrial fibrillation, previous studies have shown conflicting results [23–25].

The relationship between HDL-cholesterol and arrhythmias in our study may be attributed to the higher burden of cardiovascular risk factors in the group of patients with frequent premature complexes. HDL-cholesterol may have anti-inflammatory properties, and a link between inflammation and arrhythmias [26], such as atrial fibrillation [27, 28] and premature ventricular complexes [29] has been suggested. Therefore, lower levels of HDL-cholesterol might increase the risk of premature complexes.

The presence of intraventricular blocks on 12-lead electrocardiograms was more prevalent in participants with frequent premature atrial complexes than in those with fewer premature atrial complexes. Although blocks can be present in healthy people, they are observed more frequently in subjects with heart disease; thus, their presence might help to identify outpatients with underlying disease.

Left ventricular ejection fraction was associated with frequent premature ventricular complexes in our study. This finding is supported by prospective studies involving the general population and patients without overt structural heart disease [12,30]. They demonstrated through serial echocardiograms that higher frequencies of premature ventricular complexes were associated with a reduced left ventricular ejection fraction during follow-up [12,30]. The mechanisms by which these arrhythmias are associated with left ventricular function are not well known. Most likely the same cardiac conditions that caused left ventricular dysfunction might also have triggered the premature beats.

In our study, we found that the variables associated with premature atrial and ventricular complexes are different, except for age and BNP levels. Thus, patients with these arrhythmias should be evaluated and treated separately.

### Limitations

First, information about the medical history, lifestyle habits, and use of medications was self-reported; this could have led to misclassification of exposures. Second, the study design of a transversal study hinders the interpretation of a cause-effect relationship. Finally, the number of premature complexes may have spontaneous daily variability that contributed to reducing the power to detect significant associations.

## Conclusions

In this outpatient-based study, premature complexes were common findings on 24-hour Holter monitor recordings but were of low density. Frequent ectopic beats were associated with BNP levels > 20mg/dL and lower levels of HDL-cholesterol; left atrial enlargement and ventricular hypertrophy were also identified on the echocardiograms of these patients. The identification of frequent premature complexes on 24-hour Holter monitor recordings of outpatients in a primary public healthcare setting may be associated with uncontrolled cardiovascular risk factors that may be addressed in a primary care setting.
